# Editorial: Lifestyle behaviors and chronic diseases: pathways, interventions, knowledge and public health challenges

**DOI:** 10.3389/fmed.2026.1966213

**Published:** 2026-09-09

**Authors:** Sofia Mendes Sieczkowska, Leonardo Vidal Andreato, Guilherme Torres Vilarino, Luís Fernando Deresz, Danilo Reis Coimbra

**Affiliations:** 1 Department of Sports, School of Physical Education and Sports, Federal University of Juiz de Fora, Juiz de Fora, MG, Brazil; 2 Program of Health Promotion of Cesumar University, Maringá, Brazil; 3 Research Productivity Fellow at the Cesumar Institute of Science, Technology and Innovation, Maringá, Brazil; 4 Center for Health and Sports Sciences (CEFID), Santa Catarina State University (UDESC), Florianópolis, Brazil; 5 Faculty of Physical Education, Federal University of Rio Grande, Rio Grande, Brazil

**Keywords:** behavioral risk factors, non-communicable diseases, physical activity, sedentary behavior, unhealthy diet

Non-communicable diseases (NCDs), such as cardiovascular diseases, diabetes mellitus, and cancer, constitute approximately three-quarters of global mortality and represent a significant portion of the global health burden (1). Bernell and Howard (1) propose a conceptual shift away from rigid, disease-specific definitions and predetermined temporal criteria, advocating instead for a more parsimonious characterization of chronic conditions. The authors define “chronic” as denoting a state that is “continuing or occurring again and again for a long time” and adopt a functional perspective that emphasizes recurrence and temporal persistence over etiological specificity. Under this framework, acute episodes such as a fractured limb would be excluded from the chronic designation, whereas recurrent lower back pain or hormone-associated migraine headaches would qualify as chronic, given their episodic yet enduring nature over time.

Most of the chronic diseases can be intricately linked through modifiable lifestyle factors, where physical inactivity and poor dietary habits such as the high consumption of sugar-sweetened beverages or the expansion of modern “takeout culture” induce metabolic dysfunction long before clinical symptoms appear (Yao et al.; Waili et al.). Recent evidence underscores that traditional metrics may overlook hidden risks, such as “normal-weight obesity,” which is independently associated with systemic inflammation and increased cardiometabolic risk (Liu-Galvin et al.). Despite medical advancements, the rising prevalence of chronic conditions highlights a disconnect between scientific understanding and practical application, often exacerbated by structural barriers in primary care and suboptimal knowledge-attitude-practice (KAP) scores regarding healthy behaviors among patients (Liu, Xie et al.; Hustrini et al.).

The selected articles in this special edition provide significant insights into the Research Topic “*Lifestyle behaviors and chronic diseases: pathways, interventions, knowledge and public health challenges*” by elucidating the complex biological and psychosocial pathways between modifiable behaviors and chronic disease progression. Studies explore how specific dietary patterns such as the high consumption of sugar-sweetened beverages (Waili et al.) or the adoption of modern takeout culture (Yao et al.) contribute to an escalating global burden of malignant neoplasms and early-onset colorectal cancer (Gao, Yang et al.). Furthermore, the evidence highlights the critical role of metabolic and psychosocial determinants, demonstrating that factors like normal weight obesity (Liu-Galvin et al.) and subjective wellbeing thresholds (Iuga et al.) independently influence cardiometabolic risk and non-communicable disease (NCD) mortality. These findings emphasize the multifaceted relationships between mental health, such as depression (Wang et al.), and physiological outcomes in conditions like diabetes, metabolic syndrome (Incedal Irgat et al.), and ischemic heart disease (Aukstakalniene et al.).

In addressing interventions and public health challenges, the literature evaluates targeted management strategies and the persistent barriers to effective behavioral change. Evidence supports the efficacy of specific lifestyle interventions, including time-restricted eating, community-based physician-led hypertension management, and remote hatha yoga for chronic musculoskeletal pain (Smaira et al.; Wu et al.; Gao, Gao et al.; Andrade et al.). However, significant challenges remain regarding the translation of knowledge into practice, as evidenced by suboptimal exercise-related Knowledge-Attitude-Practice (KAP) scores in diabetic patients (Liu, Xie et al.) and barriers perceived by primary care physicians in providing physical activity counseling (Fominiene et al.). These structural and cognitive obstacles ranging from environmental disruptions in weight loss efforts (Wen et al.) to systemic inefficiencies in primary care delivery (Hustrini et al.) is essential for developing sustainable public health interventions and optimizing the management of comorbidities in diverse and underserved populations (Cano et al.; Liu, Cui et al.).

In conclusion, the findings presented in this Research Topic underscore that mitigating the global burden of non-communicable diseases requires a paradigm shift that integrates behavioral science with clinical practice. By elucidating critical pathways ranging from the inflammatory impact of normal weight obesity to the happiness threshold necessary for maximizing population health gains this Research Topic provides a robust framework for targeted prevention, knowledge gaps and health promotion.
